# MicroRNA-mediated regulation of key signaling pathways in hepatocellular carcinoma: A mechanistic insight

**DOI:** 10.3389/fgene.2022.910733

**Published:** 2022-09-02

**Authors:** Luis M. Ruiz-Manriquez, Oscar Carrasco-Morales, E. Adrian Sanchez Z, Sofía Madeline Osorio-Perez, Carolina Estrada-Meza, Surajit Pathak, Antara Banerjee, Anindya Bandyopadhyay, Asim K. Duttaroy, Sujay Paul

**Affiliations:** ^1^ Tecnologico de Monterrey, School of Engineering and Sciences, Queretaro, Mexico; ^2^ Department of Medical Biotechnology, Faculty of Allied Health Sciences, Chettinad Hospital and Research Institute (CHRI), Chettinad Academy of Research and Education (CARE), Chennai, India; ^3^ International Rice Research Institute, Manila, Philippines; ^4^ Reliance Industries Ltd., Navi Mumbai, India; ^5^ Department of Nutrition, Institute of Basic Medical Sciences, Faculty of Medicine, University of Oslo, Oslo, Norway

**Keywords:** hepatocellular carcinoma, miRNA, gene regulation, signaling pathways, therapeutics

## Abstract

Hepatocellular carcinoma (HCC) is the most common type of primary liver cancer. The molecular pathogenesis of HCC varies due to the different etiologies and genotoxic insults. The development of HCC is characterized by complex interactions between several etiological factors that result in genetic and epigenetic changes in proto-onco and/or tumor suppressor genes. MicroRNAs (miRNAs) are short non-coding RNAs that also can act as oncomiRs or tumor suppressors regulating the expression of cancer-associated genes post-transcriptionally. Studies revealed that several microRNAs are directly or indirectly involved in cellular signaling, and dysregulation of those miRNAs in the body fluids or tissues potentially affects key signaling pathways resulting in carcinogenesis. Therefore, in this mini-review, we discussed recent progress in microRNA-mediated regulation of crucial signaling networks during HCC development, concentrating on the most relevant ones such as PI3K/Akt/mTOR, Hippo-YAP/TAZ, and Wnt/β-catenin, which might open new avenues in HCC management.

## Introduction

Hepatocellular carcinoma (HCC) is the most frequent primary hepatic neoplasm with variable incidence throughout the geographical locations and represents the world’s fourth most common cause of cancer-related mortality (Kim and Viatour, 2020; Llovet et al., 2021). By 2025, the global burden of HCC-associated mortality is expected to approach 1 million per year (Siegel et al., 2017; Llovet et al., 2021). In general, HCC has a negative prognosis, given the limited therapy options, including hepatic resection and liver transplantation (Siegel et al., 2017; Singh et al., 2020; Llovet et al., 2021).

The molecular pathogenesis of HCC depends on the etiologies and genotoxic insults involved (Fardi et al., 2018; Llovet et al., 2021). Typically, activating oncogenes or inhibiting tumor suppressor genes lead to aberrations in cell signaling pathways that control cancer hallmark characteristics such as increased cell proliferation, cell fate and differentiation alterations, and resistance to programmed cell death (Juliano, 2020). Although the knowledge about the pathophysiology of HCC has recently been improved, it has yet to be implemented in advanced clinical practice (Alqahtani et al., 2019).

MicroRNAs (miRNAs) are small single-stranded RNA molecules (20–24 nucleotides) that mediate post-transcriptional gene regulation either by translational repression or mRNA degradation (Vishnoi and Rani, 2017). According to MirBase (http://www.mirbase.org) database, a total of 2,654 mature miRNAs have been reported in the *Homo sapiens* so far. Studies have shown that miRNAs are key regulators of a variety of biological activities, including cell differentiation, apoptosis, proliferation, and tumorigenesis (Paul et al., 2021; Vázquez et al., 2021; Paul et al., 2022), and their dysregulation is associated with different cancers, including HCC (Vasuri et al., 2018; Ruiz-Manriquez et al., 2021). Moreover, miRNAs are highly stable and can be quantified in several biological fluids such as blood, saliva, and urine, representing an excellent cancer biomarker (Ruiz-Manriquez et al., 2022). Intriguingly, alteration in miRNA expression profile due to certain external and internal factors potentially affects numerous signaling pathways resulting in odd changes that might lead to carcinogenesis (Leichter et al., 2017; Juliano, 2020). Hence, this review presents the current research regarding the molecular crosstalk between miRNAs and critical signal transduction networks during HCC development, focusing on the most relevant ones such as PI3K/Akt/mTOR, Hippo-YAP/TAZ, and Wnt/β-catenin.

## PI3K/Akt/mTOR pathway

The phosphatidylinositide 3-kinase (PI3K)/Akt pathway has been linked to cancer pathogenesis since its enzymatic activity was shown to be allied with viral oncoproteins (Fruman et al., 2017). It comprises several serine/threonine kinases that mediate numerous biological functions, including cell cycle progression, cell survival, migration, and protein synthesis (Alzahrani, 2019). PI3Ks are part of a family of lipid kinases that phosphorylate the 3′hydroxyl group of phosphoinositides and consist of several classes, among which the class IA PI3Ks are the most studied one and implicated in human cancers (Rahmani et al., 2020). Class IA PI3Ks are heterodimers activated downstream of receptor tyrosine kinases or RAS oncogene and contain a regulatory (p85) and a catalytic subunit (p110). Subsequently, activated PI3K triggers the production of Phosphatidylinositol-3,4,5-trisphosphate (PIP3), a crucial second messenger that in turn induces AKT (a protein kinase with pleckstrin homology domain). Afterward, AKT endorses proliferation, cellular metabolism, differentiation, angiogenesis, and apoptosis by eliciting downstream effector proteins such as the mammalian target of rapamycin (mTOR), which is key to maintaining the balance between cell proliferation and autophagy in response to cellular stress (Jiang et al., 2018; Rahmani et al., 2020).

To date, it has been well established that miRNA dysregulation is crucial in HCC development and progression. In this context, Sun et al. (2019) revealed that being a tumor suppressor, miR-1914 (poorly expressed in HCC cell lines) might hinder tumor growth and colony formation, leading to cell cycle arrest and increased apoptosis. Notably, the main target of miR-1914 is GPR39, a zinc-activated G protein-coupled receptor, which regulates HCC cell proliferation and differentiation, leading to PI3K/AKT/mTOR repression.

Likewise, Wu et al. (2020) reported that tumor sizes, tumor numbers, TNM stage, and histological grade are strongly linked with miR-660-5p expression. Furthermore, *in vitro* and *in vivo* experiments revealed that miR-660-5p could dramatically increase HCC cell proliferation, clone formation, migration, invasion, and tumorigenic potential, whereas its downregulation suppresses malignant growth. It has been proposed that epithelial cancer cells undergo an epithelial-mesenchymal transition (EMT), which is characterized by cell adhesion loss, E-cadherin suppression, acquisition of mesenchymal markers (such as N-cadherin, Vimentin, and Fibronectin), and enhanced cell motility and invasiveness (Roche, 2018). Interestingly, Wu et al. (2020) also found that miR-660-5p directly targets YWHAH, a 14-3-3 family protein that binds to phosphoserine-containing proteins to facilitate signal transduction and activates PI3K/AKT signaling pathway resulting in EMT promotion.

Yu et al. (2019) noticed that HCC tissues had considerably greater levels of miR-106b-5p than normal liver tissues. Moreover, induced miR-106b-5p could diminish the expression of FOG2, a novel inhibitor of PI3K/Akt signaling to promote the proliferation and invasion of HCC cells. In another analogous study, Yao et al. (2015) observed Metastasis-associated with Colon Cancer 1 (MACC1) gene as a novel prognostic HCC indicator that inhibited apoptosis of HCC cells by targeting the PI3K/AKT pathway**.** Intriguingly, Zhang Y. M. et al. (2020) established that miR-34a and miR-125a-5p refrained proliferation and metastasis while inducing apoptosis by suppressing the MACC1-mediated PI3K/AKT/mTOR pathway in HCC both *in vitro* and *in vivo*.

In various cancers, the oncogenic tripartite motif-containing 27 (TRIM27) protein enhances cell survival, proliferation, migration, and invasion (Zhang et al., 2018)**.** In this context, Gao et al. (2019) demonstrated that miR-30b-3p might prevent HCC cells from proliferating, migrating, and invading by downregulating TRIM27 and subsequently inactivating the PI3K/Akt pathway. Contrastingly, Du et al. (2019) noticed a significant overexpression of miR-3691-5p in HCC tissues and cell lines substantially linked to clinicopathological characteristics such as TNM stage and vascular invasion through activating PI3K/Akt signaling by targeting PTEN, and they considered this miRNA as an HCC oncomiR. Likewise, Wang et al. (2021) demonstrated that when miR-92a-3p is overexpressed, N-cadherin and Vimentin protein (two crucial markers in the transition of malignant cells from normal cells) expression levels increase, and HCC cell proliferation, migration, and invasion were stimulated, suggesting that miR-92a-3p plays a vital role in HCC cell EMT as an oncomiR. Remarkably, they also found that the PI3K/AKT/mTOR signaling pathway is activated by miR-92a-3p and induces EMT, promoting the HCC’s malignant development.

As discussed, the PI3K pathway might represent an attractive candidate for tumor therapeutic targeting. In this sense, multiple kinases in the PI3K/AKT/mTOR pathway were chosen for inhibitory activity, and the development of kinase inhibitors with improved specificity and pharmacokinetics has recently facilitated research on the PI3K pathway inhibition clinical trials. Moreover, since numerous miRNAs modulate PI3K/Akt/mTOR pathway during carcinogenesis, they could also be a promising tool for HCC management.

## Hippo-YAP/TAZ pathway

In a highly conserved manner, the Hippo-YAP/TAZ pathway modulates tissue homeostasis, organ size, cell regeneration, and growth (Samji et al., 2021), and its dysregulation has been allied with a variety of malignancies, including HCC (Xin Y. et al., 2020). A kinase cascade containing serine/threonine-protein kinase 4/3 (MST1/2), large tumor suppressor kinases (LATS) 1/2, the transcription coactivators yes-associated protein (YAP), and its paralog WW domain-containing transcription regulator protein 1 [WWTR1 or transcriptional coactivator with PDZ-binding motif (TAZ)] are critical components of this signaling pathway in mammalian cells. MST1/2 is activated by phosphorylation or trans-autophosphorylation; later, it binds to the Salvador family WW domain-containing protein 1 (SAV1) in a heterotetramer to mediate MST1/2 activation and localization to the plasma membrane. MOB 1 (monopolar spindle one-binder) aids in the recruitment of LATS1/2 to MST1/2, allowing MST1/2 to phosphorylate LATS, causing LATS autophosphorylation and activation. The linker phosphorylation sites of MST1/2 also use the striatin-interacting phosphatase and kinase complex to dephosphorylate and inactivate MST1/2, providing negative responses that limit MST activity. Upstream regulators KIBRA and Mer/NF2 soothed the Hippo kinase cascade by recruiting LATS to the plasma membrane, where Hippo/MST will activate it. Activated LATS1/2 then phosphorylate and inactivate YAP/TAZ, which leads to proteasomal decay. YAP and TAZ are not phosphorylated and hence stable when the Hippo kinase cascade is inactivated; therefore, they translocate into the nucleus and bind to transcription factors to modulate target gene expression (Liu et al., 2020). Unphosphorylated YAP penetrates the nucleus and activates oncogenes such as CYR61, AREG, AKD1, and CTGF (Zhang and Zhou, 2019).

Intriguingly, YAP and TAZ directly control miRNA biogenesis (Mori et al., 2014); while several miRNAs have been shown to target and modulate the Hippo-YAP/TAZ signaling pathway’s main components. For example, Xin Y. et al. (2020) noticed that being a transcriptional target of the Hippo-YAP/TAZ pathway miR-135b silences MST1 expression as an oncomiR, and consequently, the MST1-YAP-miR-135b axis generates a positive feedback loop in HCC advancement. Moreover, the level of miR-135b was shown to be favorably connected with HCC stages and negatively associated with HCC patient survival. These findings provide a clue by which miR-135b promotes HCC tumorigenesis through Hippo signaling pathway modulation. Guan et al. (2019) revealed that the expression of MEIS2C/D (a critical transcription factor linked to the development of human cancer) is significantly upregulated in HCC and correlated with poor prognosis. Furthermore, employing both *in vitro* and *in vivo* approaches, they demonstrated that MEIS2D enhances hepatoma cell proliferation and metastasis *via* the Hippo-YAP/TAZ signaling pathway. Interestingly, MiR-1307-3p is a key component of the MEIS2D route because MEIS2D and its synergistic molecule, PBX1, co-activated its expression. They also discovered that LATS1 is a functional target of miR-1307-3p, whose inhibition reduces YAP phosphorylation. These data imply that MEIS2D promotes HCC development *via* the miR-1307-3p/LATS1/YAP circuit. Following the same line**,** Wu et al. (2019) reported that miR-29c-3p expression was considerably reduced in HCC cell lines and tissues. Since this miRNA regulates the methylation of LATS1 by targeting DNMT3B, and aberrant methylation of LATS1 inactivates the Hippo-YAP/TAZ signaling pathway, its poor expression induces tumor growth, multiple pathologic characteristics, and shorter overall survival. However, overexpression of miR-29c-3p has also been shown to suppress HCC cell proliferation, apoptosis, migration, and tumor growth *in vivo* by negatively regulating the DNA methyltransferases 3B (DNMT3B). These findings suggest that this miRNA potentially functions as a tumor suppressor in HCC by inhibiting DNMT3B and the LATS1-associated Hippo-YAP/TAZ signaling pathway, representing a novel potential therapeutic target for HCC.

Hypoxia is a key component of the microenvironment of solid tumors, and it promotes cancer growth. Zhang B. et al. (2020) noticed that hypoxia triggers the miR-512-3p expression in HCC, and its upregulation is linked with adverse clinicopathological features, including tumor size, vascular invasion, and advanced tumor-node-metastasis phases. Moreover, LATS2 was found to be a direct functional target of miR-512-3p, and therefore, in HCC tissues, the level of miR-512-3p was negatively correlated with LATS2 expression and Hippo-YAP/TAZ signaling. Altogether, the results suggested that hypoxia-induced miR-512-3p expression inhibits the Hippo-YAP/TAZ pathway, which leads to HCC cell proliferation, migration, and invasion.

It is well established that miR-21 is strongly connected with the Hippo-YAP/TAZ signaling pathway (An et al., 2018). Recently, Hong et al. (2021) showed that miR-21-3p levels are substantially increased in HCC tissues compared to the adjacent healthy liver tissues, and the targets of this miRNA exhibited a significant association with the TGF-*β* transduction and Hippo-YAP/TAZ signaling pathway. Moreover, they demonstrated that one of the most significant targets of miR-21-3p, intranuclear SMAD7, promotes YAP1 translocation to the cytoplasm and hinders YAP1 transcription. Interestingly, YAP1 promotes SMAD7 to activate TbRI and inhibits the TGF-*β*/SMAD signal transduction; therefore, the counterbalance between SMAD7 and YAP1 significantly impacts the TGF-*β* signal transduction. This result highlighted the oncogenic role of miR-21-3p in HCC by promoting malignant phenotype progression *via* the Hippo-YAP/TAZ pathway.

Even though a number of studies have been conducted recently to identify miRNAs and their regulatory role in HCC *via* modulating Hippo-YAP/TAZ pathway, it is only the tip of the iceberg, and further research is needed to deeply understand the mechanism to develop novel therapeutics strategies against HCC.

## Wnt/β-catenin pathway

The Wnt/β-catenin is a conserved signaling axis involved in a variety of physiological settings, including differentiation, proliferation, apoptosis, migration, invasion, and tissue homeostasis (He and Tang, 2020; Zhang and Wang, 2020). Over the past years, onco or tumor suppressor miRNAs have been demonstrated to regulate HCC cell proliferation, invasion, metastasis, and drug sensitivity through modulating the key regulatory factors in the canonical Wnt/β-catenin signaling pathway.

In this milieu, Huang et al. (2020) highlighted miR-1246 as a potential factor that promotes HCC tumor formation by suppressing the expression of its target RORα. Notably, they confirmed that artificial induction of miR-1246 expression or RORα knockdown substantially augments the metastatic capacity of HCC both *in vitro* and *in vivo* through the activation of the Wnt/β-catenin pathway and epithelial-mesenchymal transition (EMT) promotion. Likewise, Xin R. Q. et al. (2020) showed that USP22, a histone-modifying enzyme principally regulated by miR-329-3p (normally downregulated in HCC), is linked to distant metastasis, poor prognosis, and high recurrence rates in HCC since it critically modulates the proliferation, metastasis, DNA repair, and stemness of tumor cells *via* modulating Wnt/β-catenin pathway**.**

C-x-C motif chemokine ligand 12 (CXCL12) is a crucial cancer immunity and angiogenesis regulator that triggers HCC progression through Wnt/β-catenin pathway regulation. Lu et al. (2019) revealed that upregulated miR-342 (which is usually poorly expressed in HCC cells) could significantly suppress the proliferation of HCC cells and increase apoptosis by targeting CXCL12 expression and subsequent inhibition of Wnt/β-catenin signaling activity. Correspondingly, the SOX family of transcription factors has emerged as modulators of canonical Wnt/β-catenin signaling (Ashrafizadeh et al., 2020). Specifically, SOX6 (downregulated in cancerous tissue, including HCC) is an anti-tumor gene that prevents cancer cells from proliferating and becoming tumorigenic (Jiang et al., 2018). Recently, Cao et al. (2021) showed that miR-19a-3p and miR-376c-3p might stimulate the Wnt/β-catenin pathway in HCC cells by targeting the SOX6. Moreover, they observed that SOX6 might bind to β-catenin and prevent it from dissociating from the transcriptional complex, preventing it from being translocated to the nucleus. Overall, this finding suggested that both miR-19a-3p and miR-376c-3p are highly expressed in HCC cells and might play a role in HCC formation by targeting SOX6 and altering the Wnt/β-catenin signaling pathway.

The protein regulator of cytokinesis 1 (PRC1) has been shown to exert an oncogenic function by promoting tumor formation, transfer, stemness, and progression of early HCC through the Wnt/β-catenin signaling pathway modulation, and its overexpression has been linked to poor HCC patient survival (Chen et al., 2016; Wang et al., 2017). Remarkably, Tang et al. (2019) demonstrated that the upregulation of miR-194 in HCC diminishes the expression of PRC1 and β-catenin accompanied by increased E-cadherin expression leading to EMT inhibition and Wnt/β-catenin signaling pathway inactivation. While in another study, WW domain-binding protein 2 (WBP2) was reported to interrelate with various WW domain-containing proteins, including WW domain-containing transcription regulator protein 1 (TAZ) and WW domain-containing oxidoreductase (WWOX), and favorably linked with the Wnt/β-catenin signaling pathway to promote downstream gene transcription, resulting in HCC progression (Chen et al., 2017). In this regard, Gao et al. (2020) proved that upregulation of miR-485-5p in HCC cells suppresses WBP2 expression and prevents Wnt/β-catenin signaling, leading to the inhibition of proliferation, migration, and invasion, as well as most significantly, suppression of tumor development *in vivo*. The authors transfected HCC cells with miR-485-5p mimic to better understand the role of miR485-5p in HCC, and they discovered that E-cadherin expression was upregulated while MMP-9, c-myc, cyclin D, and MMP-7 expression was considerably reduced. E-cadherin, also known as CDH1, is an important cancer suppressor, and its poor expression is allied with EMT, which is known to accelerate cancer cell migration and invasion.

Wilms’ tumor 1 gene (WT1) is an essential nuclear factor for organ development and cell growth. Overexpression of WT1 has been shown to be oncogenic in several types of cancers (Qi et al., 2015). Notably, it was also stated that miR-361-5p directly targets WT1 and negatively regulates its expression in HCC; moreover, its downregulation is associated with lymph node metastasis and advanced TNM stage, resulting in a poor prognosis for HCC patients. Furthermore, the effect of miR-361-5p on EMT and the WNT/β-catenin pathway was explored, and it was revealed that the overexpression of miR-361-5p hinders the N-cadherin and Vimentin expressions and promotes E-cadherin expression, inhibiting cell metastasis *via* blocking EMT as well as inactivating WNT/β-catenin pathway (Cheng et al., 2019).

In the past few years, it has been noticed that the Wnt/β-catenin pathway is abnormally activated in several types of cancer, and hence Wnt-targeted therapy has received much attention, and recently Wnt signaling has been translated to preclinical research since effective small-molecule drugs have been developed to modulate the pathway. Nevertheless, an in-depth investigation regarding the microRNA-mediated modulation of this pathway in HCC is necessary to develop advanced disease management strategies.

## Discussion

**FIGURE 1:**
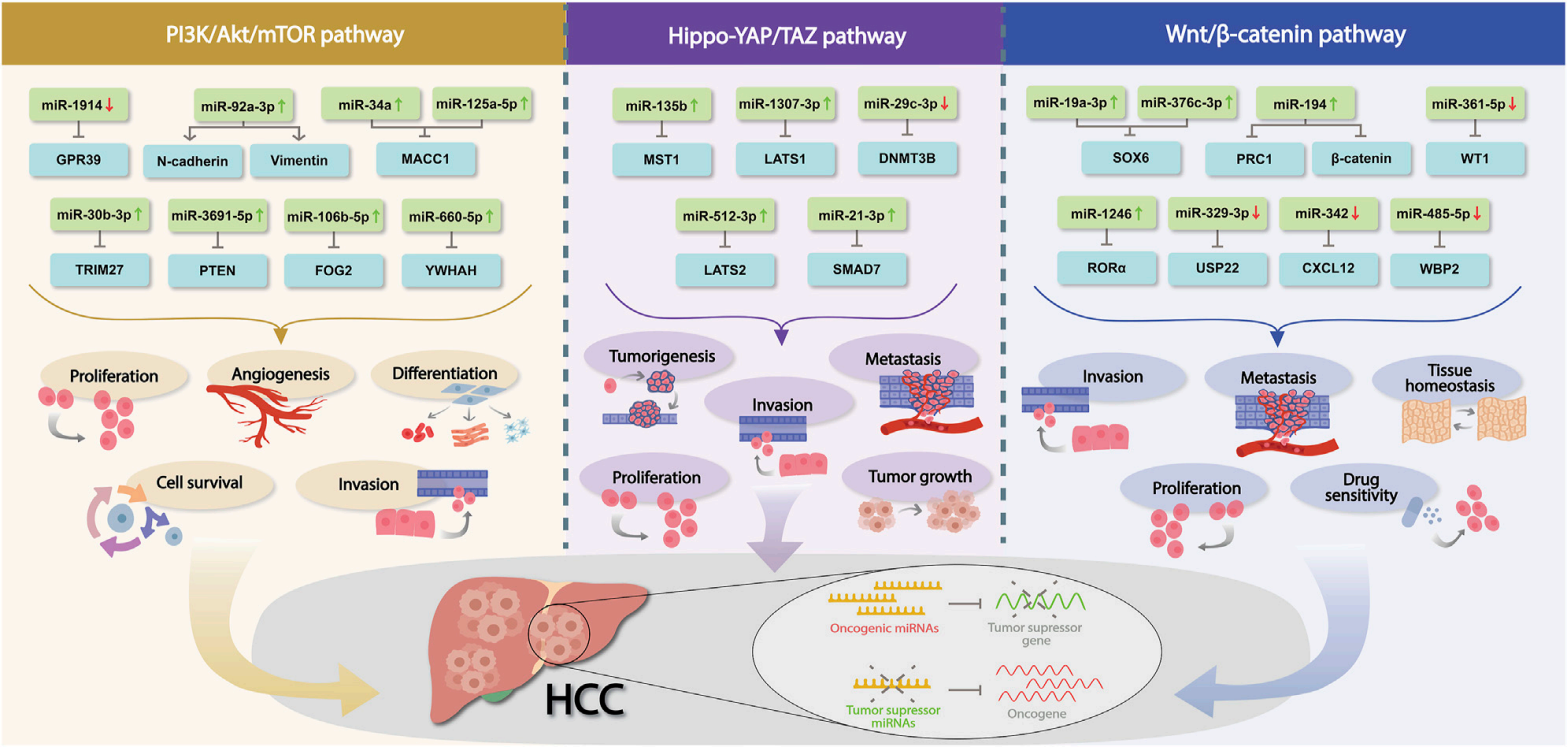
Dysregulated miRNAs in HCC involved in PI3K/Akt/mTOR, Hippo-YAP/TAZ and Wnt/β-catenin pathways. Association of a number of miRNAs in those crucial signaling pathways during HCC development, their corresponding mRNAs targets and the biological mechanism implicated are shown. Red and green arrows indicate the differential expression of each miRNA (↑ = upregulation, ↓ = downregulation).

It is well established that miRNAs are significantly involved in hepatic tumorigenicity and progression, and investigators have observed that dysregulated miRNAs promote tumorigenesis post-transcriptionally by influencing oncogenes and tumor suppressors, impacting associated canonical pathways (Figure 1). In this background, due to its crucial involvement in tumor development, metastasis, angiogenesis, stemness, and chemoresistance, PTEN/PI3K/Akt was thoroughly investigated. Indisputably, the PTEN/PI3K/Akt -signaling pathway was found to be highly dysregulated in HCC, and different miRNAs control several associated dysregulated genes. However, further investigations about miRNAs and PTEN/PI3K/Akt signaling and their molecular interactions are necessary to improve the clinical management of patients with HCC. Likewise, the hepatocyte appears to be an important cell type that is influenced by the Hippo-YAP/TAZ signaling pathway in a variety of ways. However, the explicit molecular mechanisms by which Hippo-YAP/TAZ signaling regulates multiple aspects of hepatocyte physiology and pathology remain elusive. Although a number of studies have been performed to identify miRNAs and their contribution to the Hippo-YAP/TAZ regulatory pathway during HCC development, there is still a long way to go to thoroughly understand its underlying molecular mechanism. Similarly, oncogenic or tumor suppressor miRNAs have been shown to influence HCC cell proliferation, invasion, metastasis, and drug response by targeting regulatory factors in the canonical Wnt/β-catenin signaling pathway. Notably, feedback regulation of miRNAs *via* the canonical Wnt/β-catenin signaling pathway might have a role in HCC progression; however, specific upstream regulators of miRNAs targeting this pathway must be thoroughly investigated before it reaches the clinics. Nevertheless, a greater understanding of the interaction between miRNAs and the canonical Wnt/β-catenin signaling pathway would disclose the underlying cause of HCC and aid in the development of innovative therapeutic approaches.

Additionally, significant risk factors for HCC include non-alcoholic fatty liver disease (NAFLD), chronic alcohol consumption, aflatoxin B1 exposure, hepatitis B (HBV) and C virus (HCV) infection (Fujiwara et al., 2018); and strong evidence has drawn attention to the notion that miRNAs could be critical determinants in setting these risk factors and, therefore, might play essential roles in the pathogenesis of HCC (Morishita et al., 2021). For example, some essential miRNAs are related to NAFLD pathogenesis since they perform pivotal regulatory functions of hepatic lipid metabolism (López-Sánchez et al., 2021). At the same time, the role of miRNAs in alcohol-related liver disease (ARLD) and the modulatory effects of alcohol consumption on miRNA expression have been reviewed in the past years since miRNAs can regulate the complex interplay between heavy alcohol consumption along with susceptibility to the disease (Torres et al., 2018). Moreover, in HCC pathogenesis, the alteration of miRNAs’ expression in response to xenobiotic exposure (including aflatoxin B1 exposure Balasubramanian et al., 2020); as well as their regulatory role in early HBV and HCV infection, has also been explicitly studied (Lee et al., 2017; Sartorius et al., 2019).

In recent years, significant progress has been made demonstrating miRNA regulation in various cancer, including HCC, and since several signaling pathways are substantially associated with it (Table 1), we believe it is worth writing this review describing miRNA-mediated modulation of the most relevant signaling pathways in HCC development. Undoubtedly, the interaction between miRNAs and signaling pathways in hepatic pathophysiology is complex, but new relevant information is rapidly growing, which might help to develop advanced HCC therapy.

**TABLE 1:** Differentially expressed miRNA profile in crucial signaling pathways of HCC.

miRNA	Target gene	Effect on signaling pathway	Affected biological mechanism	Function	Reference
PI3K/Akt/mTOR					
miR-1914 ↓	GPR39	Repression	Tumor growth and apoptosis	Tumor suppressor	Sun et al. (2019)
miR-660-5p ↑	YWHAH	Activation	Cell proliferation, clone formation, migration, invasion	OncomiR	Roche (2018); Wu et al. (2020)
miR-106b-5p ↑	FOG2	Activation	Cell proliferation and cell invasion	OncomiR	Yu et al. (2019)
miR-30b-3p ↑	TRIM27	Repression	Proliferation, migration, and invasion	Tumor suppressor	Gao et al. (2019)
miR-3691-5p ↑	PTEN	Activation	Vascular invasion	OncomiR	Du et al. (2019)
miR-92a-3p ↑	N-cadherin and Vimentin protein	Activation	Cell proliferation, migration, and invasion	OncomiR	Wang et al. (2021)
Hippo-YAP/TAZ					
miR-135b ↑	MST1	Activation	Cell proliferation, migration, and invasion	OncomiR	Xin et al. (2020b)
miR-1307-3p ↑	LATS1	Activation	Cell proliferation, migration, and invasion	OncomiR	Guan et al. (2019)
miR-29c-3p ↓	DNMT3B	Repression	Cell proliferation, apoptosis, migration, and tumor growth	Tumor suppressor	Wu et al. (2019)
miR-512-3p ↑	LATS2	Repression	Cell proliferation, migration, and invasion	OncomiR	Zhang et al. (2020a)
miR-21-3p ↑	SMAD7	Activation	Malignant phenotype progression	OncomiR	Hong et al. (2021)
Wnt/β-catenin					
miR-1246 ↑	RORα	Activation	Tumor growth	OncomiR	Huang et al. (2020)
miR-329-3p ↓	USP22	Activation	Proliferation, migration, invasion, DNA repair, and stemness	Tumor suppressor	Xin et al. (2020a)
miR-342 ↓	CXCL12	Repression	Cell proliferation and apoptosis	Tumor suppressor	Lu et al. (2019)
miR-19a-3p ↑ and miR-376c-3p ↑	SOX6	Activation	Cell proliferation, migration, and invasion	OncomiR	Cao et al. (2021)
miR-194 ↑	PRC1 and β-catenin	Repression	Proliferation, migration, invasion, and stemness	OncomiR	Tang et al. (2019)
miR-485-5p ↓	WBP2	Repression	Proliferation, migration, and invasion	Tumor suppressor	Gao et al. (2020)
miR-361-5p ↓	WT1	Repression	Proliferation, migration, and invasion	Tumor suppressor	Cheng et al. (2019)

## References

[B1] Alqahtani A. Khan Z. Alloghbi A. Ahmed T. S. S. Ashraf M. Hammouda D. M. (2019). Hepatocellular carcinoma: Molecular mechanisms and targeted therapies. Med. (B Aires) 55. 10.3390/MEDICINA55090526 PMC6780754 31450841

[B2] Alzahrani A. S. (2019). PI3K/Akt/mTOR inhibitors in cancer: At the bench and bedside. Semin. Cancer Biol. 59. 10.1016/J.SEMCANCER.2019.07.009 31323288

[B3] An Y. Zhang Q. Li X. Wang Z. Li Y. Tang X. (2018). Upregulated microRNA miR-21 promotes the progression of lung adenocarcinoma through inhibition of KIBRA and the Hippo signaling pathway. Biomed. Pharmacother. 108, 1845–1855. 10.1016/J.BIOPHA.2018.09.125 30372890

[B4] Ashrafizadeh M. Taeb S. Hushmandi K. Orouei S. Shahinozzaman M. Zabolian A. (2020). Cancer and SOX proteins: New insight into their role in ovarian cancer progression/inhibition. Pharmacol. Res. 161. 10.1016/J.PHRS.2020.105159 32818654

[B5] Balasubramanian S. Gunasekaran K. Sasidharan S. Jeyamanickavel Mathan V. Perumal E. (2020). MicroRNAs and xenobiotic toxicity: An overview. Toxicol. Rep. 7, 583–595. 10.1016/J.TOXREP.2020.04.010 32426239 PMC7225592

[B6] Cao X. Zhang J. Apaer S. Yao G. Li T. (2021). microRNA-19a-3p and microRNA-376c-3p promote hepatocellular carcinoma progression through SOX6-mediated wnt/β-catenin signaling pathway. Int. J. General Med. 14, 89. 10.2147/IJGM.S278538 PMC7812052 33469348

[B7] Chen J. Rajasekaran M. Xia H. Zhang X. Kong S. N. Sekar K. (2016). The microtubule-associated protein PRC1 promotes early recurrence of hepatocellular carcinoma in association with the Wnt/β-catenin signalling pathway. Gut 65, 1522–1534. 10.1136/GUTJNL-2015-310625 26941395 PMC5036256

[B8] Chen S. Wang H. Huang Y. F. Li M. L. Cheng J. H. Hu P. (2017). WW domain-binding protein 2: An adaptor protein closely linked to the development of breast cancer. Mol. Cancer 16. 10.1186/S12943-017-0693-9 PMC5518133 28724435

[B9] Cheng Y. Qiu L. He G. L. Cai L. Peng B. J. Cao Y. L. (2019). MicroRNA-361-5p suppresses the tumorigenesis of hepatocellular carcinoma through targeting WT1 and suppressing WNT/β-cadherin pathway. Eur. Rev. Med. Pharmacol. Sci. 23, 8823–8832. 10.26355/EURREV_201910_19277 31696469

[B10] Du W. Zhang X. Wan Z. (2019). miR-3691-5p promotes hepatocellular carcinoma cell migration and invasion through activating PI3K/Akt signaling by targeting PTEN. Onco Targets Ther. 12, 4897–4906. 10.2147/OTT.S208127 31417285 PMC6593750

[B11] Fardi M. Solali S. Farshdousti Hagh M. (2018). Epigenetic mechanisms as a new approach in cancer treatment: An updated review. Genes Dis. 5, 304–311. 10.1016/J.GENDIS.2018.06.003 30591931 PMC6303480

[B12] Fruman D. A. Chiu H. Hopkins B. D. Bagrodia S. Cantley L. C. Abraham R. T. (2017). The PI3K pathway in human disease. Cell 170, 605. 10.1016/J.CELL.2017.07.029 28802037 PMC5726441

[B13] Fujiwara N. Friedman S. L. Goossens N. Hoshida Y. (2018). Risk factors and prevention of hepatocellular carcinoma in the era of precision medicine. J. Hepatol. 68, 526–549. 10.1016/J.JHEP.2017.09.016 28989095 PMC5818315

[B14] Gao D. Zhou Z. Huang H. (2019). miR-30b-3p inhibits proliferation and invasion of hepatocellular carcinoma cells *via* suppressing PI3K/Akt pathway. Front. Genet. 10, 1274. 10.3389/FGENE.2019.01274/BIBTEX 31921311 PMC6923265

[B15] Gao J. Dai C. Yu X. Yin X. B. Zhou F. (2020). microRNA-485-5p inhibits the progression of hepatocellular carcinoma through blocking the WBP2/Wnt signaling pathway. Cell Signal 66. 10.1016/J.CELLSIG.2019.109466 31706018

[B16] Guan L. Li T. Ai N. Wang W. He B. Bai Y. (2019). MEIS2C and MEIS2D promote tumor progression *via* Wnt/β-catenin and hippo/YAP signaling in hepatocellular carcinoma. J. Exp. Clin. Cancer Res. 38, 1–14. 10.1186/S13046-019-1417-3/FIGURES/7 31623651 PMC6796342

[B17] He S. Tang S. (2020). WNT/β-catenin signaling in the development of liver cancers. Biomed. Pharmacother. 132, 110851. 10.1016/J.BIOPHA.2020.110851 33080466

[B18] Hong Y. Ye M. Wang F. Fang J. Wang C. Luo J. (2021). MiR-21-3p promotes hepatocellular carcinoma progression *via* SMAD7/YAP1 regulation. Front. Oncol. 11, 303. 10.3389/FONC.2021.642030/BIBTEX PMC7982593 33763375

[B19] Huang J. L. Fu Y. P. Gan W. Liu G. Zhou P. Y. Zhou C. (2020). Hepatic stellate cells promote the progression of hepatocellular carcinoma through microRNA-1246-RORα-Wnt/β-Catenin axis. Cancer Lett. 476, 140–151. 10.1016/J.CANLET.2020.02.012 32061951

[B20] Jiang W. Yuan Q. Jiang Y. huang L. Chen C. Hu G. (2018). Identification of Sox6 as a regulator of pancreatic cancer development. J. Cell. Mol. Med. 22, 1864. 10.1111/JCMM.13470 29369542 PMC5824410

[B21] Juliano R. L. (2020). Addressing cancer signal transduction pathways with antisense and siRNA oligonucleotides. Nar. Cancer 2(3):zcaa025. 10.1093/NARCAN/ZCAA025 33015625 PMC7520847

[B22] Kim E. Viatour P. (2020). Hepatocellular carcinoma: Old friends and new tricks. Exp. Mol. Med. 52 (12). 1898-1907. 10.1038/s12276-020-00527-1 33268834 PMC8080814

[B23] Lee C. H. Kim J. H. Lee S.-W. (2017). The role of MicroRNA in pathogenesis and as markers of HCV chronic infection. Curr. Drug Targets 18, 756–765. 10.2174/1389450117666160401125213 27033188

[B24] Leichter A. L. Sullivan M. J. Eccles M. R. Chatterjee A. (2017). MicroRNA expression patterns and signalling pathways in the development and progression of childhood solid tumours. Mol. Cancer 16 (1), 1–17. 10.1186/S12943-017-0584-0 28103887 PMC5248531

[B25] Liu Y. Wang X. Yang Y. (2020). Hepatic Hippo signaling inhibits development of hepatocellular carcinoma. Clin. Mol. Hepatology 26, 742. 10.3350/CMH.2020.0178 PMC7641559 32981290

[B26] Llovet J. M. Kelley R. K. Villanueva A. Singal A. G. Pikarsky E. Roayaie S. (2021). Hepatocellular carcinoma. Nat. Rev. Dis. Prim. 7 (1 7), 1–28. 10.1038/s41572-020-00240-3 33479224 PMC13537433

[B27] López-Sánchez G. N. Dóminguez-Pérez M. Uribe M. Chávez-Tapia N. C. Nuño-Lámbarri N. (2021). Non-alcoholic fatty liver disease and microRNAs expression, how it affects the development and progression of the disease. Ann. Hepatology 21, 100212. 10.1016/J.AOHEP.2020.04.012 32533953

[B28] Lu C. Jia S. Zhao S. Shao X. (2019). MiR-342 regulates cell proliferation and apoptosis in hepatocellular carcinoma through Wnt/β-catenin signaling pathway. Cancer Biomark. 25, 115–126. 10.3233/CBM-192399 31006667 PMC13082419

[B29] Mori M. Triboulet R. Mohseni M. Schlegelmilch K. Shrestha K. Camargo F. D. (2014). Hippo signaling regulates microprocessor and links cell-density-dependent miRNA biogenesis to cancer. Cell 156, 893–906. 10.1016/J.CELL.2013.12.043 24581491 PMC3982296

[B30] Morishita A. Oura K. Tadokoro T. Fujita K. Tani J. Masaki T. (2021). MicroRNAs in the pathogenesis of hepatocellular carcinoma: A review. Cancers (Basel) 13, 1–29. 10.3390/CANCERS13030514 PMC7866004 33572780

[B31] Paul S. Bravo Vázquez L. A. Reyes-Pérez P. R. Estrada-Meza C. Aponte Alburquerque R. A. Pathak S. (2022). The role of microRNAs in solving COVID-19 puzzle from infection to therapeutics: A mini-review. Virus Res. 308. 10.1016/J.VIRUSRES.2021.198631 PMC8590742 34788642

[B32] Paul S. Ruiz-Manriquez L. M. Ledesma-Pacheco S. J. Benavides-Aguilar J. A. Torres-Copado A. Morales-Rodríguez J. I. (2021). Roles of microRNAs in chronic pediatric diseases and their use as potential biomarkers: A review. Archives Biochem. Biophysics 699, 108763. 10.1016/J.ABB.2021.108763 33460581

[B33] Qi X. W. Zhang F. Wu H. Liu J. L. Zong B. G. Xu C. (2015). Wilms’ tumor 1 (WT1) expression and prognosis in solid cancer patients: A systematic review and meta-analysis. Sci. Rep. 5. 10.1038/SREP08924 PMC4352850 25748047

[B34] Rahmani F. Ziaeemehr A. Shahidsales S. Gharib M. Khazaei M. Ferns G. A. (2020). Role of regulatory miRNAs of the PI3K/AKT/mTOR signaling in the pathogenesis of hepatocellular carcinoma. J. Cell. Physiology 235, 4146–4152. 10.1002/JCP.29333 31663122

[B35] Roche J. (2018). The epithelial-to-mesenchymal transition in cancer. Cancers (Basel) 10, 52. 10.3390/CANCERS10020052 PMC5836084 29462906

[B36] Ruiz-Manriquez L. M. Estrada-Meza C. Benavides-Aguilar J. A. Ledesma-Pacheco S. J. Torres-Copado A. Serrano-Cano F. I. (2021). Phytochemicals mediated modulation of microRNAs and long non-coding RNAs in cancer prevention and therapy. Phytother. Res. 36 (2), 705–729. 10.1002/PTR.7338 34932245

[B37] Ruiz-Manriquez L. M. Ledesma Pacheco S. J. Medina-Gomez D. Uriostegui-Pena A. G. Estrada-Meza C. Bandyopadhyay A. (2022). A brief review on the regulatory roles of MicroRNAs in cystic diseases and their use as potential biomarkers. Genes (Basel) 13. 10.3390/GENES13020191 PMC8872411 35205236

[B38] Samji P. Rajendran M. K. Warrier V. P. Ganesh A. Devarajan K. (2021). Regulation of hippo signaling pathway in cancer: A MicroRNA perspective. Cell. Signal. 78, 109858. 10.1016/J.CELLSIG.2020.109858 33253912

[B39] Sartorius K. Makarova J. Sartorius B. An P. Winkler C. Chuturgoon A. (2019). The regulatory role of MicroRNA in hepatitis-B virus-associated hepatocellular carcinoma (HBV-HCC) pathogenesis. Cells 8, 1504. 10.3390/CELLS8121504 PMC6953055 31771261

[B40] Siegel R. L. Miller K. D. Jemal A. (2017). Cancer statistics, 2017. CA Cancer J. Clin. 67, 7–30. 10.3322/CAAC.21387 28055103

[B41] Singh G. Yoshida E. M. Rathi S. Marquez V. Kim P. Erb S. R. (2020). Biomarkers for hepatocellular cancer. World J. Hepatology 12, 558. 10.4254/WJH.V12.I9.558 PMC7522562 33033565

[B42] Sun L. Wang L. Chen T. Yao B. Wang Y. Li Q. (2019). microRNA‐1914, which is regulated by lncRNA DUXAP10, inhibits cell proliferation by targeting the GPR39-mediated PI3K/AKT/mTOR pathway in HCC. J. Cell. Mol. Med. 23, 8292. 10.1111/JCMM.14705 31576658 PMC6850956

[B43] Tang H. Zhao H. Yu Z. Y. Feng X. Fu B. S. Qiu C. H. (2019). MicroRNA-194 inhibits cell invasion and migration in hepatocellular carcinoma through PRC1-mediated inhibition of Wnt/β-catenin signaling pathway. Dig. Liver Dis. 51, 1314–1322. 10.1016/J.DLD.2019.02.012 30948333

[B44] Torres J. L. Novo-Veleiro I. Manzanedo L. Suárez L. A. MacÍas R. Laso F. J. (2018). Role of microRNAs in alcohol-induced liver disorders and non-alcoholic fatty liver disease. World J. Gastroenterology 24, 4104. 10.3748/WJG.V24.I36.4104 PMC6158486 30271077

[B45] Vasuri F. Visani M. Acquaviva G. Brand T. Fiorentino M. Pession A. (2018). Role of microRNAs in the main molecular pathways of hepatocellular carcinoma. World J. Gastroenterology 24, 2647. 10.3748/WJG.V24.I25.2647 PMC6034147 29991871

[B46] Vázquez B. Becerril M. Hernández M. Carmona L. Padilla A. Phylactou A. (2021). The emerging role of MicroRNAs in bone diseases and their therapeutic potential. Molecules 27, 211. *Page 211* 27. 10.3390/MOLECULES27010211 35011442 PMC8746945

[B47] Vishnoi A. Rani S. (2017). MiRNA biogenesis and regulation of diseases: An overview. Methods Mol. Biol. 1509, 1. 10.1007/978-1-4939-6524-3_1 27826912

[B48] Wang L. Cui M. Qu F. Cheng D. Yu J. Tang Z. (2021). MiR-92a-3p promotes the malignant progression of hepatocellular carcinoma by mediating the PI3K/AKT/mTOR signaling pathway. Curr. Pharm. Des. 27, 3244–3250. 10.2174/1381612827666210612054156 34126886

[B49] Wang Y. Shi F. Xing G. H. Xie P. Zhao N. Yin Y. F. (2017). Protein regulator of cytokinesis PRC1 confers chemoresistance and predicts an unfavorable postoperative survival of hepatocellular carcinoma patients. J. Cancer 8, 801–808. 10.7150/JCA.17640 28382142 PMC5381168

[B50] Wu H. Zhang W. Wu Z. Liu Y. Shi Y. Gong J. (2019). miR-29c-3p regulates DNMT3B and LATS1 methylation to inhibit tumor progression in hepatocellular carcinoma. Cell Death Dis. 10 (2), 48. 10.1038/s41419-018-1281-7 30718452 PMC6362005

[B51] Wu Y. Zhang Y. Wang F. Ni Q. Li M. (2020). MiR-660-5p promotes the progression of hepatocellular carcinoma by interaction with YWHAH *via* PI3K/Akt signaling pathway. Biochem. Biophysical Res. Commun. 531, 480–489. 10.1016/J.BBRC.2020.07.034 32807493

[B52] Xin R. Q. Li W. B. Hu Z. W. Wu Z. X. Sun W. (2020a). MiR-329-3p inhibits hepatocellular carcinoma cell proliferation and migration through USP22-Wnt/β-Catenin pathway. Eur. Rev. Med. Pharmacol. Sci. 24, 9932–9939. 10.26355/EURREV_202010_23204 33090397

[B53] Xin Y. Yang X. Xiao J. Zhao W. Li Y. Lu L. (2020b). MiR-135b promotes HCC tumorigenesis through a positive-feedback loop. Biochem. Biophysical Res. Commun. 530, 259–265. 10.1016/J.BBRC.2020.07.008 32828296

[B54] Yao Y. Dou C. Lu Z. Zheng X. Liu Q. (2015). MACC1 suppresses cell apoptosis in hepatocellular carcinoma by targeting the HGF/c-MET/AKT pathway. Cell. Physiology Biochem. 35, 983–996. 10.1159/000369754 25660117

[B55] Yu L. X. Zhang B. L. Yang M. Y. Liu H. Xiao C. H. Zhang S. G. (2019). MicroRNA-106b-5p promotes hepatocellular carcinoma development *via* modulating FOG2. Onco Targets Ther. 12, 5639. 10.2147/OTT.S203382 31406464 PMC6642636

[B56] Zhang B. Huang L. Tu J. Wu T. (2020a). Hypoxia-induced placenta-specific microRNA (miR-512-3p) promotes hepatocellular carcinoma progression by targeting large tumor suppressor kinase 2. Onco Targets Ther. 13, 6073. 10.2147/OTT.S254612 32612368 PMC7323795

[B57] Zhang S. Zhou D. (2019). Role of the transcriptional coactivators YAP/TAZ in liver cancer. Curr. Opin. Cell Biol. 61, 64–71. 10.1016/J.CEB.2019.07.006 31387016

[B58] Zhang Y. Feng Y. Ji D. Wang Q. Qian W. Wang S. (2018). TRIM27 functions as an oncogene by activating epithelial-mesenchymal transition and p-AKT in colorectal cancer. Int. J. Oncol. 53, 620. 10.3892/IJO.2018.4408 29767249 PMC6017157

[B59] Zhang Y. M. Wu Q. M. Chang L. Y. Liu J. C. (2020b). miR-34a and miR-125a-5p inhibit proliferation and metastasis but induce apoptosis in hepatocellular carcinoma cells *via* repressing the MACC1-mediated PI3K/AKT/mTOR pathway. Neoplasma 67, 1042–1053. 10.4149/NEO_2020_191019N1062 32484698

[B60] Zhang Y. Wang X. (2020). Targeting the Wnt/β-catenin signaling pathway in cancer. J. Hematol. Oncol. 13 (1 13), 1–16. 10.1186/S13045-020-00990-3 33276800 PMC7716495

